# Complement System: Promoter or Suppressor of Cancer Progression?

**DOI:** 10.3390/antib9040057

**Published:** 2020-10-25

**Authors:** Margot Revel, Marie V. Daugan, Catherine Sautés-Fridman, Wolf H. Fridman, Lubka T. Roumenina

**Affiliations:** Team Inflammation, Complement and Cancer, Centre de Recherche des Cordeliers, INSERM, Sorbonne Université, Université de Paris, F-75006 Paris, France; margot.revel@gmail.com (M.R.); dauganmarie7@hotmail.fr (M.V.D.); catherine.sautes-fridman@sorbonne-universite.fr (C.S.-F.); herve.fridman@crc.jussieu.fr (W.H.F.)

**Keywords:** complement system, cancer, immune infiltrate, tumor microenvironment, tumor growth, anaphylatoxins

## Abstract

Constituent of innate immunity, complement is present in the tumor microenvironment. The functions of complement include clearance of pathogens and maintenance of homeostasis, and as such could contribute to an anti-tumoral role in the context of certain cancers. However, multiple lines of evidence show that in many cancers, complement has pro-tumoral actions. The large number of complement molecules (over 30), the diversity of their functions (related or not to the complement cascade), and the variety of cancer types make the complement-cancer topic a very complex matter that has just started to be unraveled. With this review we highlight the context-dependent role of complement in cancer. Recent studies revealed that depending of the cancer type, complement can be pro or anti-tumoral and, even for the same type of cancer, different models presented opposite effects. We aim to clarify the current knowledge of the role of complement in human cancers and the insights from mouse models. Using our classification of human cancers based on the prognostic impact of the overexpression of complement genes, we emphasize the strong potential for therapeutic targeting the complement system in selected subgroups of cancer patients.

## 1. Introduction

The 21st century was marked by a change in the paradigm of tumor perception. Scientists have established the important role of the immune system and inflammation in cancer development and especially the role of T cells. This concept was not only useful as an academic discovery but it also led to development of several novel treatments, as well as anti-immune checkpoint therapies (anti-PD1/PDL1, anti-CTLA4) that were rewarded by the Nobel prize of 2018. Immune cells have the ability to infiltrate tumors and form with other untransformed cells the tumor microenvironment (TME) [1]. The TME can impact positively or negatively the patient’s outcome, depending on its composition [2]. The recruitment of immune cells inside the tumor is achieved thanks to the vascular network that also allows the recruitment of the components of the complement system. The complement system is often forgotten or underestimated, but it is a powerful inflammatory cascade and, as a part of innate immunity, it fully belongs to the TME [3]. The complement system is a set of more than thirty cell-bound or soluble proteins that can come inside the tumor via the circulation but also that can be produced by the tumor cells themselves and the infiltrated immune cells. The complement system is mostly described by its functions related to immunity but, recently, several papers attribute it non-immune functions as angiogenesis, organ development and regeneration or also neuroprotection [4,5].

In this review, we will focus on the different functions of this very complex system and how they can influence patient’s outcome, depending of the cancer types or the pathway activated.

## 2. The Complement System

**Figure 1:**
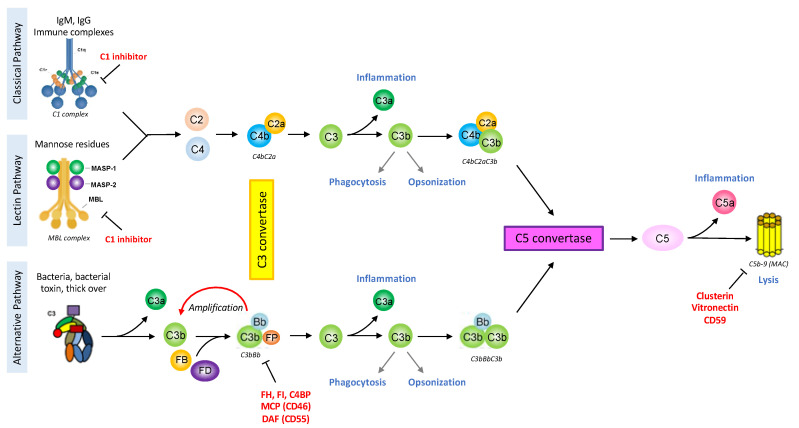
The complement system schematic summary. The classical pathway is activated by the binding of the C1 complex to immunoglobulins or endogenous ligand. The lectin pathway is analogous to the classical one but its activation is triggered by the fixation of the MBL-MASP complex to the pathogen surface. The alternative pathway is spontaneously initiated by the tick-over mechanism and can be amplified in case of recognition of an unprotected surface by complement regulators. These pathways will lead to the formation of the C3 convertase, an enzymatic complex able to cleave C3 into the anaphylatoxin C3a and C3b. The assemblage of a C3b molecule to the C3 convertase is at the origin of the C5 convertase. The C5 molecule can then be cleaved into the anaphylatoxin C5a and C5b, the latter initiating the terminal pathway. The complement cascade culminates with the formation of the multimeric Membrane Attack Complex (MAC, C5b-9) leading to cell activation or death. The complement system is very powerful in triggering inflammation, phagocytosis, opsonization or also lysis, therefore it is tightly regulated at each step by soluble regulators (C1 inhibitor, Factor I (FI), C4 Binding Protein (C4BP), Factor H (FH), Properdin (FP) clusterin, vitronectin) or membrane proteins (Complement Receptor 1 (CD35, CR1), Membrane Cofactor Protein (CD46, MCP), Decay acceleration Factor (CD55, DAF), CD59). The figure is created with BioRender.com.

The first description of the complement system in 1890 assigned it antimicrobial functions [6]. However, due to its composition and the plurality of its actions, the complement system was very difficult to study and progress was dependent on the technologies available. Since the 1950s, with the development of protein chromatography and electrophoresis, data have never stopped to accumulate [7]. Complement is one of the first lines of defense against pathogens or stressed host cells, and can be triggered, depending on the activator, by three different pathways: classical, lectin and alternative. They lead to the formation of C3 and C5 convertases and the common terminal pathway (Figure 1). The complement proteins interact in a highly regulated proteolytic cascade to opsonize pathogens, induce inflammation, interact with cells of adaptive immunity, and maintain homeostasis [4]. The complexity of the complement system is not only due to its composition or its numerous functions (immune or non-immune) but also its ability to act extracellularly or intracellularly.

### 2.1. Complement Activating Pathways

The classical pathway is activated after the binding of the C1 complex to its targets that can be an antigen-antibody (IgG or IgM) immune complex or an apoptotic cell. The C1 complex is composed of C1q molecules and two different serine protease C1r and C1s [8]. The hexagonal arrangement of the protein platforms (hexameric organization of the IgGs bound to an antigen, or the structure of IgM) is critical for the C1 complex activation [9,10]. The lectin pathway is activated by the recognition of sugar residues through a complex that is structurally and functionally very similar to the classical pathway [11]. The MBL molecule (mannan-binding-lectin), collectins or ficolins resemble C1q and are associated with several serine proteases (MASP1, MASP2 and MASP3), just like the C1 complex. These pathways will lead to the cleavage of C2 into C2a and C2b and C4 into C4a and C4b. C4b and C2a form the classical pathway C3 convertase (C4bC2a), an enzymatic complex able to cleave C3 into the anaphylatoxin C3a and C3b. The specificity of the alternative pathway, in physiology, is to be constitutively activated at low level, thanks to the tick-over mechanism: a spontaneous hydrolysis of a C3 molecule [12], used as a surveillance system. Although properdin could serve as alternative pathway initiator [13], this pathway usually does not need a trigger. This allows the formation of C3(H_2_O), a bio-active form of C3, structurally and functionally similar to C3b. C3(H_2_O) can bind Factor B (FB) that could be cleaved by Factor D (FD) into Bb and Ba fragments, forming the fluid phase alternative pathway C3 convertase: C3(H_2_O)Bb, cleaving C3 into the anaphylatoxin C3a and opsonin C3b. In absence of activation the newly formed C3b will be hydrolyzed. In presence of an activating surface, C3b will bind covalently, recruit FB and FD, thereby forming the alternative C3 convertase (C3bBb). This mechanism allows the establishment of an amplification loop to increase the C3 cleavage and hence the C3b concentration at the target surface (pathogen or stressed cell).

### 2.2. Complement Effector Pathways

Covalent deposition of C3b on the target cell by any of the three pathways induces opsonization, allowing the elimination of the pathogen/stressed cell by phagocytosis [12]. When a certain density of C3b is reached, the molecule of C3b is assembled to the C3 convertase and leads to the formation of the C5 convertase (classical: C4bC2aC3b, or alternative: C3bBbC3b). This triggers the terminal pathway with the generation of the C5a and C5b fragments, and finally the last reaction with the formation of the membrane attack complex (MAC: composed by the molecules C5b, C6, C7, C8 and C9) [4].

Complement activation allows the formation of three different effectors: Opsonins (C3b, C4b and C1q) that can bind the target cell surface and promote its clearance. Anaphylatoxins (C3a and C5a) are released in the circulation and have an important role to induce inflammation and activate cells expressing anaphylatoxin receptors (C3aR, C5aR1/C5aR2). C5aR1 and C5aR2 seem to have opposite effects, especially in the tumor context [14]. MAC formed by the association of C5b molecule with C6 that acquire the ability to interact with lipid bilayer, the C7 and C8 molecules bind C5b and insert into lipid bilayer, then several molecules of C9 polymerize creating the lytic pore, causing membrane permeabilization, cell activation and/or the cell death [15].

### 2.3. Complement Regulators

Complement is a very powerful system that needs to be tightly regulated. To maintain the balance between complement activation on pathogens or altered host cells and inhibition on intact host cells, several regulators control each step of the proteolytic cascade (Table 1) [16].

### 2.4. Complement Complexity

The complexity of complement is not only due to its composition or its numerous functions (immune or non-immune) but also due to its ability to act extracellularly or intracellularly. Indeed, although complement is often described as a set of plasma proteins mostly produced by the liver and working in the extracellular compartment, recent studies showed a potential intracellular role of its proteins, especially in T cells. In these cells, intracellular C3 and C5 can be cleaved into C3a and C5a, impacting cell metabolism and homeostasis, the induction of a Th1 and CTL response but the contraction of the T-cell response [17].

Furthermore, overactivation or deficiency of complement can trigger different pathologies, related to inflammation, coagulation, abnormal immune response, or abnormal cell clearance. A deficiency of some complement components can lead to an increased susceptibility to infections or auto-immune diseases [5]. Complement overactivation is classically associated with kidney diseases such as the atypical Hemolytic Uremic Syndrome (aHUS) and C3 Glomerulopathies [18], but also plays a role in pathologies such as multiple sclerosis [19], age-related macular degeneration [20], sickle cell disease [21], or schizophrenia [22], as well as with cancer [23].

## 3. Complement and Cancer

The complement components are mostly produced by the liver, but it is important to note that both tumor and stromal cells also have the ability to produce complement proteins. Thus, their concentration inside the tumor is both due to the contribution of the systemic compartment and the local production by the different cell types.

**Figure 2:**
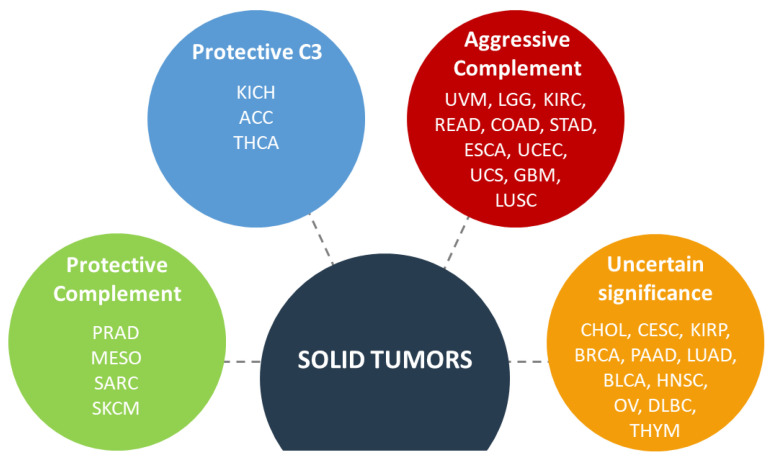
Impact of the complement gene expression on the survival of patients with solid tumors. ACC, adrenocortical carcinoma; BLCA, bladder carcinoma; BRCA, invasive breast carcinoma; CESC, cervical squamous carcinoma; CHOL, cholangiocarcinoma; COAD, colon adenocarcinoma; DLBC, diffuse large B cell lymphoma; ESCA, esophageal carcinoma; GBM, glioblastoma; HNSC, head and neck squamous cell carcinoma; KICH, kidney chromophobe carcinoma; KIRC, kidney renal clear cell carcinoma; KIRP, kidney renal papillary cell carcinoma; LGG, lower grade glioma; LUAD, lung adenocarcinoma; LUSC, lung squamous carcinoma; MESO, mesothelioma; OV, ovarian serous cystadenocarcinoma; PAAD, pancreatic adenocarcinoma; PRAD, prostate adenocarcinoma; READ, rectum adenocarcinoma; SARC, sarcoma; SKCM, skin cutaneous melanoma; STAD, stomach adenocarcinoma; THCA, thyroid carcinoma; THYM, thymoma; UCEC, uterine corpus endometrial carcinoma; UCS, uterine carcinosarcoma; UVM, uveal melanoma. The figure is created with BioRender.com.

Analysis of complement gene expression in thirty different cancers revealed consistent patterns of expression: a high expression of genes coding for classical and alternative pathway; high expression of regulators and a low expression of the lectin pathway and the terminal pathway genes [3]. These data suggest that the tumor could benefit from the early complement proteins but then sets up brakes to avoid any deleterious effector functions. This study is in line with previous observations of an abnormal expression of complement proteins in different type of cancer, especially C1 complex [23], C3 [24], C4 [25], C5 [24], C3aR [24], C5aR1 [24], FB [26], FH [27], FI [28], CD46 [29], CD55 [30], CD59 [30]. Bioinformatic analysis of the prognostic impact of the complement genes allowed to classify cancers in four groups: protective complement (concomitant occurrence of favorable prognosis associated with high expression of complement genes), protective C3 (favorable prognosis found only for high C3 expression but not for the other genes), aggressive complement (concomitant occurrence of poor prognosis, associated with high expression of complement genes) and uncertain significance of complement (when no particular pattern is observed) [3] (Figure 2).

It seems that it is not possible to reach a general conclusion as for the pro or anti-tumor role of the complement system. This context-dependent action is reflected by numerous studies, sometimes contradictory, about complement and cancer. 

### 3.1. Activation of Complement in the Tumor Microenvironment

In a tumor context, it is not well understood how the complement system is activated, but several models show that the classical and alternative pathway components are found at a higher concentration in the tumor microenvironment [31]. The tumor cells develop the ability to produce a set of complement proteins and to hijack other proteins produced by host cells, to trigger the complement activation. In clear cell Renal Cell Carcinoma (ccRCC), tumor cells produce C1r and C1s and use C1q secreted by macrophages, in order to form a functional C1 complex and activate the classical pathway in IgG-containing deposits [23] or on cell-bound pentraxin 3 (PTX3) [32]. The hypothesis that the complement system could be activated by immunoglobulins is not new. In 1996, in thyroid carcinoma the presence of IgG, together with C4d, C3d and C5-positive staining suggested tumor-specific classical pathway activation [33]. In non-small cell lung cancer (NSCLC), IgM deposits are observed, pointing to a potential binding of the IgM to the neoantigens present at the tumor cell surface and a triggering of the classical pathway activation [34]. Furthermore, in human urothelial urinary bladder cancer, 50% of tumors with high levels of IgG antibodies also display C1q [35]. Interestingly, in this high IgG and C1q positive tumors, almost 40% also present membranous staining with an anti-C3a detecting antibody. This study suggests the possibility that the activation of the classical pathway could be favorable for the patient’s survival [35]. However, recent studies revealed that in lung and renal cancers the activation of the classical pathway by intratumoral immunoglobulins is associated with poor prognosis [23,34]. Interestingly, C3aR and C5aR are likely expressed at the surface of most cell types in a tumor [36] suggesting that C3a and C5a could be used by the tumor cells to promote tumor growth. The activation of the complement system leads to the regulation of the immune system but also amplification of tumor cell invasiveness by acting on proliferation, migration, and epithelial-mesenchymal transition [37].

### 3.2. Role of Complement on Tumor Immunity

In the last decade, there was an increasing interest in the role of complement in cancer progression. Even if the classical functions of this system are to favor cell killing, its role in cancer appears to be mostly pro-tumoral. The first clue was the slower tumor growth in case of C3, C4 or C5aR deficiency in a TC1 cancer mouse model [38].

Mouse models were, and still are, very useful to study the mechanisms of action and impact of complement on immune cells in the case of cancer. Nevertheless, mouse models yielded some contradictory findings, stressing the context-dependent role of complement in cancer. C3 and C3a are reported to play an important role in cancer progression. Indeed, several studies using syngeneic mouse models (melanoma, breast, and colon cancer [39], or hepatocellular carcinoma [40]) go in the same direction. The C3 expressed notably by immune cells [41] enhances tumor growth by promoting an immunosuppressive environment. Its cleavage product C3a supports the recruitment of C3aR+ macrophages, and perturbation of C3a/C3aR axis disrupts immune infiltration, slowing tumor growth [41]. C3a also promotes T-cell apoptosis, inhibition of T-cell proliferation, inhibition of dendritic cell maturation, increasing of the macrophage and MDSC (myeloid-derived suppressor cells) recruitment, leading to a reduction in the number of CD8+ T cells [41]. A recent study suggests that C3b could impact tumorigenesis during chronic skin inflammation, in cutaneous squamous cell carcinoma (cSCC) model, but independently of C3aR or the terminal pathway (C5a/C5aR1/C5aR2 and MAC generation), likely by influencing tumor associated macrophages [42]. Another report suggests that in colorectal cancer, only the C5a/C5aR1 axis and not C3, could play an important role in the modulation of tumor immunity, by recruiting MDSC and promoting tumorigenesis [43]. Tumor cells can produce C3 molecules, and this production leads to PD-L1 antibody treatment resistance [44]. This mechanism is described in a colon cancer mouse model and goes through a modulation of the tumor associated macrophages response in order to repress anti-tumor immunity, via the C3a-C3aR-PI3Kγ way. These studies highlight again how the complement response can be context—dependent and even contradictory depending of the model and on the cancer types. The multitude of mechanisms by which C3 affects tumor growth is important and must be taken into consideration in the process of development of potential therapeutics [45].

The other anaphylatoxin, C5a, was largely studied too, for its role in tumor progression. Syngeneic mouse models (cervical cancer, lung cancer, or breast cancer) revealed that C5a and its receptor C5aR1 are involved in the recruitment of MDSC. C5a can amplify their capacities to produce reactive oxygen species (ROS) and reactive nitrogen species (RNS) creating an environment favorable for the suppression of the anti-tumor CD8+ T-cell mediated response [38]. In addition, the MDSC recruitment favors the generation of Treg and Th2 response that can suppress the anti-tumor CD8+ T cells [46], but also mediates production of the immunomodulators ARG1, CTLA-4, IL-6, IL-10, LAG3 and PDL1 [47].

An important regulator of the intratumoral levels of C5a is PTX3; this molecule interacts with C1q and FH to modulate the local complement activation. PTX3 is described as an oncosuppressor in mouse models, inhibiting complement activation via FH recruitment, limiting the production of C5a and CCL2 (a pro-inflammatory chemokine) and avoiding the recruitment of tumor-promoting macrophages. The PTX3 deficiency leads to a chronic complement-mediated inflammation, favoring spontaneous skin carcinoma development in a mice model [48]. On the other hand, in human cancer context (ccRCC), a high expression of PTX3 is associated with lower survival rates [32]. The authors explain it by the ability of PTX3 to activate the classical pathway, leading to the production of the pro-inflammatory C3a and C5a. The role of PTX3 as an anti- or pro-tumoral molecule is not yet well understood, but is context-dependent and may be different between mice and human. Interestingly, in ccRCC the complement system is activated via the classical pathway [23], but due to the presence of CD59 regulators [32], the activation is limited and does not go to the C5b-9 lytic pore formation. Therefore, tumor cells could benefit from a complement system activation, until a certain point. 

This context-dependent role of the complement system is also true for the pro-inflammatory C3a and C5a molecules that can have anti-tumor functions and appear to be crucial for a good tumor response to radiotherapy [49]. In a carcinogen-induced cSCC model, C5a has an anti-tumoral impact [42], while in a virus-induced mouse model of cSCC, C5a has pro-tumoral impact, with the specificity to be generated independently of complement. The reason behind this phenomenon is unknown. One possible explanation could be linked to the level of locally generated C5a. Indeed, in a syngeneic lymphoma mouse model, the tumor cells producing low levels of C5a are more susceptible to apoptosis and less proliferative, leading to a smaller tumor size. These tumors are associated with an increase infiltration of granulocytes and macrophages and finally an increase IFNγ production by CD4+ and CD8+ T cells in lymph nodes and spleen [50]. However, tumors with high levels of C5a present an accelerated tumor growth with less CD4+ and CD8+ T cells, in the tumor, the spleen and the tumor-draingin lymph nodes. This concentration-dependent impact of C5a requires further exploration in different cancer contexts. 

The complement system modulates the T-cell response in a tumor context but a role of complement proteins on the B cell response was also recently described. To be efficient, the chemotherapy has to induce a specific subset of B cells, the ICOS-L+ cells (Inducible T-cell Co-Stimulator Ligand) that express the Complement Receptor 2 (CR2). The interaction of C3 fragments with CR2 promotes the ICOS-L+ B cell generation. On the over hand, the over-expression of CD55 (a complement regulator) inhibits the complement activation and prevents the formation of ICOS-L+ B cell [51]. In this context, the activation of the classical pathway through the binding of the C1 complex and immunoglobulins produced by B cells, though, has not been investigated. 

### 3.3. Impact of Complement on Tumor Cells

The tumor cells have the ability to produce complement proteins that can stimulate tumor growth directly, independently of the cascade activation. Tissue staining and transcriptomic analysis reveal that in many cancers types the presence of complement proteins is associated with a worse outcome for the patient.

#### 3.3.1. Native Proteins

C1q is a multitasking protein that has a strong direct impact on tumor cells, positively and negatively affecting their biology. In human prostate, breast, cancer or neuroblastoma, anti-tumor role of C1q is described, as an apoptosis inducer. C1q activates WWOX, a tumor suppressor gene, then the phosphorylated form of WOX1 accumulates in nuclei and sends anti-proliferative and pro-apoptotic signals [52,53]. This function is also described in ovarian cancer. By using its globular domains, C1q induces apoptosis via TNF-α (Tumor Necrosis Factor) and Fas [54]. In contrast, in melanoma, C1q favors proliferation and migration of tumor cells, increases metastasis, and decreases survival [55]. These data on C1q highlight the possible context-dependent action in different types of cancer. 

FH is mostly studied on lung and cSCC. In addition to protecting tumor cells from complement-mediated cytotoxicity, FH promotes cell migration [56]. However, FH deficient mice develop spontaneous hepatic tumors, suggesting an essential role of FH to control unwanted complement activation in the liver to avoid a complement-mediated chronic inflammation [57].

In cSCC, knock-down of C1s, C1r, FB, FH and FI exerts similar effects on the tumor growth. These proteins seem to be implicated in the promotion of the tumor cell proliferation, migration, and survival, via the activation of the signaling pathways PI3K and Erk 1/2 [28,56]. In ccRCC, tumor cells also produce a large spectrum of complement proteins [34], but whether they exert any function within the cells as for the cSCC remains unknown. Several types of tumors express complement proteins that seem to be produced by tumor cells, except for C1q (which usually comes from macrophages) [24]. The most logical suggestion is that complement proteins will be secreted in the tumor microenvironment and will activate the cascade, by hijacking C1q from the macrophages, as we found in ccRCC [23]. Nevertheless, another hypothesis is also possible: these proteins could remain within the cell and form an intracellular complement, or a “complosome” as suggested already for the T cells [58]. Although provocative and controversial [4,59], this hypothesis needs experimental verification, since compelling evidence suggests that at least C3 and C5 can be cleaved within the T cells, modulating their functions. If proven possible, intracellular and extracellular complements could act together to promote/control tumor growth. Moreover, some of the complement proteins are multitasking effectors, with functions outside of the complement cascade [55], which adds to the diversity of the complement proteins actions in cancer.

#### 3.3.2. Activation Fragments

The recognition of the anaphylatoxins by their receptors (C3aR, C5aR1 and C5aR2) present at the surface of some tumor cells leads to the activation of the signaling pathways PI3K, Erk 1/2 and AKT. The activation of these pathways favors the proliferative, survival and invasive properties of tumor cell [60]. The ability of ovarian or lung tumor cells to produce these anaphylatoxins suggests a possible autocrine activation of the cells [61]. In addition to the impact in cell proliferation, an increase of the cell migration is also observed through the C3a-C3aR recognition in melanoma [39] or through the role of C5a in development of metastasis. Indeed, in mouse models, C5 deficiency drastically decreases the hepatic metastasis in colorectal cancer [62] and C5aR facilitates the lung metastasis in breast cancer [46]. This impact on cancer metastasis can be explained by another function of C5a. The C5a-C5aR axis directly impacts the tumor cell cytoskeleton, the cells gain in motility and release some metalloproteinases (MMP) [63]. The MMP are well known to contribute to the cell migration, invasion, and metastasis. Furthermore, MMP can activate the complement system by interaction with the globular domains of C1q [64].

The anaphylatoxins are not the only complement proteins involved in cancer progression. Most frequently in a tumor context, the complement activation is stopped before the formation of the MAC. Nevertheless, even when the lytic pore is assembled, the tumor cell could escape from the lysis by activating the PI3K, AKT, Erk1/2, p70 S6 kinase signaling pathways. This results in an inhibition of the apoptosis, and finally favors the tumor progression [65].

### 3.4. Role of the Complement on Angiogenesis

Neo-angiogenesis is critical for the tumor growth, by supplying tumor cells with oxygen and nutriments. This parameter is directly linked to the tumor aggressiveness [66]. Recently, a new role of C1q, independently of complement activation, was described. Subcutaneously injected cancer cells form tumors, which in the C1q deficient mice present a disrupted vasculature architecture that could be linked to VEGF-C (vascular endothelial growth factor C) expression [23,55]. This function is, though, also context-dependent, because in a spontaneous model of breast cancer, C1q-/- mice have enhanced neoangiogenesis and hence, bigger tumors [53]. These roles were recently described and still not well understood. The role of the anaphylatoxins C3a and C5a in angiogenesis is better characterized. In a mouse model, a C3 deficiency induces, just like C1q, an alteration of the vasculature architecture, in connection with VEGF expression [67]. Additionally, C5a promotes the migration, proliferation, and vessel formation by endothelial cells [68]. The pro or anti-angiogenic role of complement is controversial. Indeed a mouse model of mammary carcinoma, it was shown that C3 activation can impair the angiogenesis [69] as well as C1q [53].

**Figure 3:**
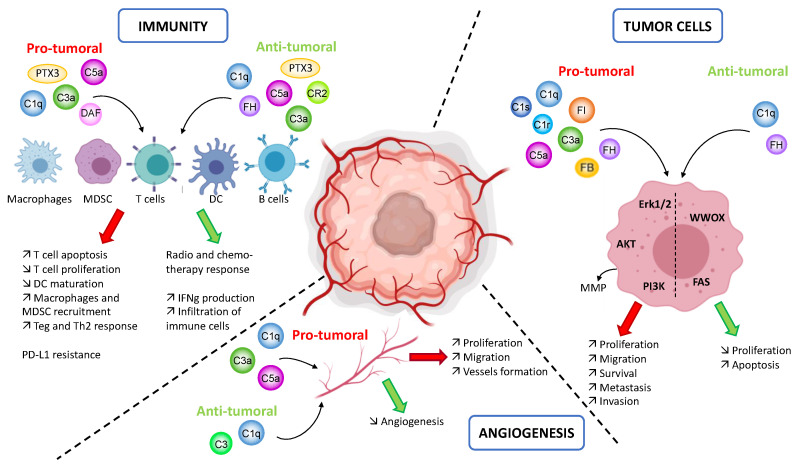
Overview of the complement functions in the context of cancer. In a tumor context, the complement system may impact the immunity, angiogenesis and the phenotype of the tumor cells. **Immunity**: the same complement proteins may impact several immune cells positively or negatively depending of the model or cancer type. The pro-tumoral activity is mostly involved in the recruitment of immune cells and in the suppression of their anti-tumoral response. The anti-tumoral activity is linked to an improvement of the response to therapy and an increase in the immune cells infiltration. **Angiogenesis**: depending on the model, the complement proteins may promote or hamper tumor growth by favoring or inhibiting the neoangiogenesis. **Tumor cells phenotype**: by modulating the signaling pathways Erk1/2, AKT and PI3K the complement proteins stimulate tumor growth. In other models, the modulation of other signaling pathway (WWOX and FAS) by complement proteins favor the apoptosis of tumor cells. The figure is created with BioRender.com.

To conclude, the complement system is involved in the key processes of the tumor progression: immunity, angiogenesis and tumor cell proliferation and spreading. Depending of the cancer types and the complement molecule, various pro- and anti-tumoral functions are described (Figure 3). This multitude of actions is a perfect example of the complexity of the complement system that we need to understand better.

### 3.5. Complement Biomarkers in Patients with Cancer

In cancer, complement components can be up-regulated or down-regulated (Table 2). Their overexpression is found at each step of the complement cascade: C1 complex (C1q, C1s), MBL complex (MBL, MASP2), alternative pathway (FH, C3), anaphylatoxins (C3a, C3a desArg, C5a, C3aR, C5aR1) and regulators (CD46, CD55, CD59). The down-regulation of complement proteins and particularly complement regulators is reported inside certain tumors, especially in ovarian cancer. Tumor cells develop several mechanisms to escape from MAC formation while benefiting from the complement activation (production of anaphylatoxins, C1q, etc.). Killing by MAC is prevented in general by overexpression of regulators and down-regulation of the terminal complement components, forming the C5b-9 complex. This appears as a general pattern, observed in transcriptomic profiles of numerous cancer types [3]. Indeed, the lack of terminal complement molecules (such as C7) would prevent the formation of the MAC [70]. MAC blockage can also be triggered by the Heat shock protein 90 (Hsp90). This protein directly interacts with the C9 molecules, are sequestered, preventing the polymerization and formation of the MAC [71].

The modifications of expression are not only found locally inside the tumor but also in the systemic compartment. This characteristic is important for cancer patients and could be helpful in the future to classify the patient and adapt the treatment with a blood test. Even if this perspective is interesting, it is important to note that an increase or decrease of protein expression in the systemic compartment does not necessarily mean that a similar modification occurs locally inside the tumor. The correlation between the situation inside the tumor and in the plasmatic compartment has to be studied to better understand the actions of complement proteins in a tumor context.

## 4. Therapeutic Aspects

The bioinformatic analysis, even if it needs biological confirmation, shows us that the tumors are not all equal in the context of complement activation. This has to be considered for the development of new complement-affecting anti-cancer therapy [24].

### 4.1. Complement Inhibitors

Considering all the data, the complement inhibition and specifically the anaphylatoxins inhibition could be a promising treatment for patients with certain cancers. Currently, the only complement inhibitors approved are acting at the level of C5, such as Eculizumab [96]. It allows complement activation but without the formation of the C5a anaphylatoxin and the membrane attack complex. On the other hand, it appeared that to have an effective response to treatment like chemotherapy and radiotherapy, a low level of complement activation is needed [49,51]. Another complement blocking agent is PMX53 that can block the C5aR1 and allow a reduction of the tumor size, in lung and melanoma mouse models. This molecule also reduces the metastasis in pancreatic model. Nevertheless, these positive effects are not universal and are dependent on the cancer type [24]. As we discussed earlier, the complement activation sets an immunosuppressive microenvironment, so it will be interesting to combine anti-complement therapy with immune checkpoint inhibitors, to reverse this effect [97]. The involvement of C1q in several anti-tumor functions and the potential of the enhancement of the complement-mediated cytotoxicity (strong enough to form the MAC and to kill tumor cells) could be interesting for potential therapeutics. To improve the C1q activation, a new generation of therapeutic antibodies is currently being developed, as IgG hexamers [98].

### 4.2. Therapeutic Inhibition of C5aR1 on Tumor Cells Versus on Immune Cells

The anaphylatoxins receptors are present at the surface of the immune cells and at the surface of some tumor cells. C5a can interact with two different receptors, C5aR1 and C5aR2, with opposite functions. C5aR1 seems to have pro-tumoral role, whereas C5aR2 has more limited impact but tends to modulate the tumor growth [14].

A common mechanism of action of the C5a-C5aR1 axis is to induce an immunosuppressive microenvironment by recruiting cells that will inactivate the effector T cells. Even if the immune checkpoint inhibitors (anti-PD1/PD-L1) have the ability to reinvigorate the exhausted effector T cells, they cannot reverse the immunosuppressive environment set up, explaining why these drugs are not working equally between patients [99]. The positive effect of combined therapy was first confirmed in mouse lung, melanoma, and colon cancer models [97,100]. Since 2018, a clinical trial in lung and liver cancers using C5aR1 and PD-L1 inhibitors is in progress (Avdoralimab plus Durvalumab: STELLAR-001 clinical study, NCT03665129) [101]. The concept is to block C5aR1, expressed on subsets of MDSC and neutrophils, unleashing thus the anti-tumor activities of the T cells and NK cells. The first results of this clinical trial are encouraging [101]: reduction of the tumor growth, of the metastatic capacity and increase of patient’s survival.

Even if C3aR and C5aR seem to be interesting targets for cancer treatment, it is important to recall that depending on the cancer their actions are not the same. Indeed, the study of the literature highlights cancer types with pro-tumor effects of C3aR/C5aR and others, with anti-tumor effects. The lung, colon, ovarian or breast cancer are often described as cancer with aggressive complement, implicated in the tumor development [102]. In parallel in these same cancers, the importance of a small amount of C3aR/C5aR seems to be necessary for a good response to radiotherapy [49]. Overall, for tumor cells C3aR/C5aR is needed for the growth, but these molecules are also needed for a good immune response as well as T-cell activation [103,104]. A treatment involving the C3aR/C5aR blocking must be well thought out, it is necessary to find an equilibrium between the positive impact that can have on tumor cells but without completely shutting down the T-cell response.

### 4.3. Complement Activation-Enhancing Therapeutic Antibodies

Monoclonal antibody (mAb)-based immunotherapy has shown promising results. Especially the anti-CD20 molecules in the context of hematological tumors. Their benefit is due in part to their ability to activate complement-dependent cytotoxicity through their Fc part [105]. In this review, we were focused on the impact of the complement system in solid tumors, but it is important to note that it plays also a role in hematological malignancies and especially on the response to therapy. The treatment of such tumors is with anti-CD20 [106], anti-CD52 [107] and anti-CD38 [108]. To be efficient these therapies are based on the mechanisms of complement-dependent cytotoxicity and complement-dependent cell-mediated phagocytosis [109]. However, in this type of cancers there often occurs complement deficiencies [110] with overexpression of complement regulators [111]. These data highlight one more time the high ability of the tumor cells to adapt their microenvironment for their development. In vitro, binding of submaximal C1q promotes complement-dependent cytotoxicity (CDC) of B cells opsonized with anti-CD20 mAbs Ofatumumab or Rituximab. Even if Rituximab allows complement activation via C1q binding, the amount of C3b deposition at the cell surface is not enough to generate the MAC, contrary to Ofatumumab, which is more effective to induce CDC [112].

It has been demonstrated that a single amino acid substitution in the IgG-Fc domain favors the formation of IgG hexamers, called HexaBodies [98]. These molecules bind more efficiently C1q [9], induce a strong complement activation and the CDC response even in presence of low level of C9 molecule [113]. Furthermore, very recent research showed that a hetero-hexamerization is possible, two m-Ab (anti-CD20 and anti-CD37) can cooperate to bind C1q and synergize their response to induce a superior CDC [114].

Another strategy is to use bi-specific antibodies. Such antibodies can recognize two different epitopes, for example one tumor-specific and the other one targeting complement membrane regulator. Bi-specific antibodies were recently developed to induce the complement cytotoxicity, by the recognition of the properdin (the positive regulator of the alternative pathway, able to stabilize the C3 convertase at the cell surface) and the EGFR (Epidermal growth factor receptor) [115]. Even if this antibody induces an increase of C3b deposition, the large presence of complement inhibitors at the tumor cell surface protects the tumor cell against the complement cytotoxicity. Another bi-specific antibody was engineered to target HLA-class I and CD55and showed an increase of C3c deposition on colorectal cancer, compared to the antibody alone or a mix of the two [116]. This technology uses the properties of C1q to work more effectively when the density of epitope is higher. The bispecific Ab technology has led to several inventions as well as the creation of a modular bi-specific platform: one unique arm recruits C1q, associated with multiple other arms able to recognize B cells, T cells or also bacteria. This platform could be applicable for diverse pathologies (infection to cancer) [117].

These new ways to engineer antibodies open doors for very interesting combined therapies.

### 4.4. Activation Versus Inhibition of Complement in Cancer

The available data have to be taken with caution, as most of it comes from mouse models, which are not always in line with the human pathology. For example, mouse models of melanoma [39], sarcomas [48] and liver [57] cancer show that complement overactivation favors tumor growth, while the transcriptomic analysis of human cancers positions them in the group, where the complement activation is potentially anti-tumoral. Detailed studies in patient cohorts will be critical to resolve this contradiction.

It is very important to always evaluate the harm-benefit ratio. The different cancer types are not equivalent and, even inside the same type of cancer, the patients are not equal and a good treatment for one patient can be inefficient or even deleterious for others. The obvious risk of using complement inhibitors is that the killing of the tumor cells by the cascade, triggered by anti-tumor neoantigens antibodies, will be impaired. This mechanism could be operating in some contexts, such as the case of anti-FH antibodies in NSCLC [118]. Cancers, in which complement inhibition could be undesirable could be the ones with a positive correlation between the IgG and C1q deposits and a prolonged survival. It is likely that in these types of cancer C5b-9 deposits will be present and will be able to kill the tumor cell. This can be achieved either by the natural history of the tumor progression or after the action of certain drugs. Complement activating anti-tumor cells antibodies will be beneficial in such cancers as well. It is difficult to predict which patients could benefit from this approach. If our classification of the cancers based on the concomitant overexpression of complement genes and their correlation with prognosis is considered, the tumors in the complement protective group (Figure 2) should fall into this category. We still need experimental validation of this concept and staining for complement in these types of tumors. Interestingly, it is already known that in sarcoma and melanoma the presence of tertiary lymphoid structures, containing IgG-producing B cells, in the human tumors correlates with a favorable outcome and response to checkpoint inhibitors [119]. Whether complement plays a role in this process remains to be defined.

On the other part of the spectrum are the tumors in which complement overexpression correlates with poor prognosis [24], and in which complement activation was experimentally proven to be associated with poor prognosis, such as ccRCC [32], lung cancer [120] or potentially the gliomas. In these cases, complement inhibition may be beneficial for the patients. In these patients, application of therapeutic antibodies with enhanced complement activating capacity may be dangerous, because the cancer cells are very well adapted to resist to complement-mediated killing and benefit from the chronic inflammation. At least in ccRCC, the IgG deposits on tumor cells trigger the local complement activation without MAC formation.

The results of the STELLAR-001 clinical trial (anti-C5aR1 Avdoralimab + anti-PD-L1 Durvalumab) will be of great interest. Positive or negative results in lung and liver cancers may not necessarily be applicable to other cancers, for which separate studies have to be performed. Interestingly, liver cancer (HCC) belongs to the “Protective complement” group (in optimal cutoff) (Figure 2), where the concomitant overexpression of complement genes is rather associated with a favorable outcome, while in the NSCLC complement activation, measured at protein level, is associated with poor prognosis [120]. Staining for complement is needed in HCC in order to understand its mechanism of action in this type of cancer.

In any case, due to the pleiotropic effects of complement, it is complicated at present to predict whether and to what extend complement modulation will be beneficial or harmful in patients with cancer. This is, therefore, an exciting area of research with a great future potential.

## 5. Conclusions

Recent discoveries in the complement system are very challenging. The complement proteins act everywhere in the body, extracellularly and intracellularly, they have functions related to the complement activation or independent of it. This new understanding just adds to the already known complexity and plurality of this system. The actions of complement in the tumor context are diverse: action on the immune cells, on the cancer cells and also action on angiogenesis. What this review highlights is the complexity of these functions that can sometimes be opposite depending on the cancer type. It appears that the complement activation mostly has pro-tumor effects, but its complete inhibition may not be always desirable. Indeed, a good response to treatment (chemotherapy or radiotherapy) needs low level of complement activation for the setting of an anti-tumor immunity.

To conclude, it appears clear that the complement system has to be considered for the development of new therapies. However, a large spectrum of complement modulators are entering the market, opening numerous possibilities in cancer therapy [3].

## Figures and Tables

**Table 1:** Complement regulators. The complement system is well regulated at different steps by various soluble or membranous proteins.

Regulated Steps	Regulators	Soluble or Membranous
C1 complex/MBL complex	C1 inhibitors	Soluble
C3 convertases C5 convertases	Factor H (FH)	Soluble
	CFHRs (1 to 5)	Soluble
	Properdin (FP)	Soluble
	C4 Binding Protein (C4BP)	Soluble
	Factor I (FI)	Soluble
	Membrane cofactor proteins (MCP/CD46)	Membranous
	Decay acceleration factor (DAF/CD55)	Membranous
	Complement receptor 1 (CR1)	Membranous
MAC	CD59	Membranous
	Clusterin	Soluble
	Vitronectin	Soluble

**Table 2:** Overview of the different modifications of expression of complement proteins in a tumor context. The upper part of the table summarizes the overexpression of complement proteins described in different cancer types. The overexpression of complement proteins occurs at each step of the complement cascade: classical pathway, lectin pathway, alternative pathway, anaphylatoxins, MAC and regulators. The lower table summarizes the down-regulation of complement proteins described in the literature. The down-regulation of complement proteins involves the classical and alternative pathway, and complement regulators.

**Overexpression**			
**Molecule**	**Type of Cancer**	**Mechanism of Action**	**Ref.**
C1q	Glioblastoma	Plasma: increased C1q in the sera of patients in comparison with healthy controls.	[72]
C1s	Lung cancer	Plasma: increased levels of C1s in plasma of lung cancer patient in comparison with controls	[73]
	Prostate cancer	Tumor: Up-regulation of C1s expression in prostate tumors compared to matched normal prostate tissues	[74]
C4	Lung cancer	Plasma: elevated C4 levels in cancer patients in comparison to control group	[75]
C4a	Papillary thyroid cancer	Plasma: increased C4a in the sera of patients in comparison with healthy controls.	[76]
C4d	Lung cancer	Bronchial fluid: Higher levels of C4d in cancer patients than patients with control group.	[77]
	Lung cancer	Plasma: Higher levels of C4d in cancer patients than patients with benign nodules.	[25]
C3	Lung cancer	Plasma: elevated C3 levels in cancer patients in comparison to control group	[75]
	Neuroblastoma	Plasma: elevated C3 levels in cancer patients in comparison to healthy donors	[78]
	Pancreatic ductal adenocarcinoma	Tumor: Higher levels of C3 protein in cancerous tissues than in adjacent normal pancreatic tissues	[79]
	Pancreatic cancer	Tumor: Higher levels of C3 protein in cancerous tissues than in normal pancreatic tissues	[80]
C3a	Esophageal cancer	Plasma: Higher C3a levels in patients than healthy donors	[81]
C3a desArg	Breast cancer	Plasma: Higher C3a desArg level in patients than healthy donors	[82]
C5a	Non-small cell lung cancer	Plasma: Higher C3a levels in patients than healthy donors	[47]
C5aR1	Gastric cancer	Tumor: higher expression of C5aR1 in gastric tumoral tissues than in adjacent non-tumoral tissues	[83]
FH	Squamous lung cancer	Plasma: Up-regulation of FH in uranium exposed miners in comparison with exposed miners without lung disease	[84]
	Lung cancer	Bronchoalveolar lavage: Higher concentration of factor H in lung cancer patients than controls	[85]
	Cutaneous squamous cell carcinoma	Tumor: FH is more expressed in invasive cSCC than normal skin or in situ cSCC.	[56]
	Bladder cancer	Urines: FH and FH related protein are markers for bladder cancer	[86,87]
FI	Cutaneous squamous cell carcinoma	Tumor: FI is more expressed in invasive cSCC than normal skin or in situ cSCC.	[28]
C9	Squamous cell lung cancer	Plasma: C9 and its fucosylated form are significantly higher in SQLC patients, as compared to healthy control	[88]
CD46	Colon cancer	Tumor: CD46 is higher in colon cancer tissues compared with normal adjacent colon tissues	[89]
CD55	Colon cancer	Tumor: CD55 is higher in colon cancer tissues compared with normal adjacent colon tissues	[89]
CD59	Colon cancer	Tumor: CD59 is higher in colon cancer tissues compared with normal adjacent colon tissues	[89]
MASP2	Ovarian tumor	Tumor: MASP2 gene expression is higher with ovarian cancer compared with controls	[90]
MBL	Colon tumor	Plasma: MBL2 levels increases in patients compared to healthy blood donors.	[91]
	Ovarian tumor	Tumor: MBL2 gene expression is higher with ovarian cancer compared with controls	[90]
**Underexpression**			
C1s	Ovarian cancer	Tumor: Down-regulation of C1s mRNA in ovarian tumor vs healthy control	[92]
	Ovarian cancer	Tumor: Down-regulation of C1s expression in stage III serous ovarian carcinoma compared to normal tissue	[93]
	Lung cancer	Tumor: decrease expression in lung tumor tissues in comparison with peritumoral tissues	[73]
C4BP	Ovarian cancer	Tumor: Down-regulation of C4BPA mRNA in ovarian tumor vs healthy control	[92]
C7	Ovarian cancer	Tumor: Down-regulation of C7 mRNA in ovarian tumor vs healthy control	[92]
FB	Glioblastoma	Plasma: decreased level of FB in GBM	[72]
FI	Gastric cancer	Plasma: FI is significantly lower in gastric cancer sera compared to normal sera. Declining expression with the advanced pTNM stage from stage I to IV of gastric cancer patients	[94]
FH	Colon cancer	Plasma: Decrease in FH protein level in the serum of colorectal cancer patients vs. normal control	[95]
	Ovarian cancer	Tumor: Down-regulation of FH mRNA in ovarian tumor vs healthy control	[92]
CD55	Ovarian cancer	Tumor: Lower expression of CD55 in ovarian cancer than in control	[30]

## References

[1] Fridman W.H., Pagès F., Sautès-Fridman C., Galon J. (2012). The immune contexture in human tumours: Impact on clinical outcome. Nat. Rev. Cancer.

[2] Fridman W.H., Zitvogel L., Sautès-Fridman C., Kroemer G. (2017). The immune contexture in cancer prognosis and treatment. Nat. Rev. Clin. Oncol..

[3] Roumenina L.T., Daugan M.V., Petitprez F., Sautès-Fridman C., Fridman W.H. (2019). Context-dependent roles of complement in cancer. Nat. Rev. Cancer.

[4] Merle N.S., Church S.E., Fremeaux-Bacchi V., Roumenina L.T. (2015). Complement System Part I—Molecular Mechanisms of Activation and Regulation. Front. Immunol..

[5] Merle N.S., Noe R., Halbwachs-Mecarelli L., Fremeaux-Bacchi V., Roumenina L.T. (2015). Complement System Part II: Role in Immunity. Front. Immunol..

[6] Buchner: Zur Nomenklatur der schutzenden Eiweisskorper—Google Scholar. https://scholar.google.com/scholar_lookup?journal=Centr+Bakteriol+Parasitenk.&title=Zur+Nomenklatur+der+schutzenden+Eiweisskorper.&author=H+Buchner&volume=10&publication_year=1891&pages=699-701&.

[7] Sim R.B., Schwaeble W., Fujita T. (2016). Complement research in the 18th–21st centuries: Progress comes with new technology. Immunobiology.

[8] Gaboriaud C., Thielens N.M., Gregory L.A., Rossi V., Fontecilla-Camps J.C., Arlaud G.J. (2004). Structure and activation of the C1 complex of complement: Unraveling the puzzle. Trends Immunol..

[9] Sharp T.H., Boyle A.L., Diebolder C.A., Kros A., Koster A.J., Gros P. (2019). Insights into IgM-mediated complement activation based on in situ structures of IgM-C1-C4b. Proc. Natl. Acad. Sci. USA.

[10] Ugurlar D., Howes S.C., de Kreuk B.-J., Koning R.I., de Jong R.N., Beurskens F.J., Schuurman J., Koster A.J., Sharp T.H., Parren P.W.H.I. (2018). Structures of C1-IgG1 provide insights into how danger pattern recognition activates complement. Science.

[11] Garred P., Genster N., Pilely K., Bayarri-Olmos R., Rosbjerg A., Ma Y.J., Skjoedt M.-O. (2016). A journey through the lectin pathway of complement-MBL and beyond. Immunol. Rev..

[12] Ricklin D., Reis E.S., Mastellos D.C., Gros P., Lambris J.D. (2016). Complement component C3—The “Swiss Army Knife” of innate immunity and host defense. Immunol. Rev..

[13] Kemper C., Atkinson J.P., Hourcade D.E. (2010). Properdin: Emerging roles of a pattern-recognition molecule. Annu. Rev. Immunol..

[14] Nabizadeh J.A., Manthey H.D., Panagides N., Steyn F.J., Lee J.D., Li X.X., Akhir F.N.M., Chen W., Boyle G.M., Taylor S.M. (2019). C5a receptors C5aR1 and C5aR2 mediate opposing pathologies in a mouse model of melanoma. FASEB J..

[15] Tegla C.A., Cudrici C., Patel S., Trippe R., Rus V., Niculescu F., Rus H. (2011). Membrane Attack by Complement: The Assembly and Biology of Terminal Complement Complexes. Immunol. Res..

[16] Noris M., Remuzzi G. (2013). Overview of Complement Activation and Regulation. Semin. Nephrol..

[17] West E.E., Kunz N., Kemper C. (2020). Complement and human T cell metabolism: Location, location, location. Immunol. Rev..

[18] Blanc C., Togarsimalemath S.K., Chauvet S., Le Quintrec M., Moulin B., Buchler M., Jokiranta T.S., Roumenina L.T., Fremeaux-Bacchi V., Dragon-Durey M.-A. (2015). Anti-factor H autoantibodies in C3 glomerulopathies and in atypical hemolytic uremic syndrome: One target, two diseases. J. Immunol..

[19] Michailidou I., Willems J.G.P., Kooi E.-J., van Eden C., Gold S.M., Geurts J.J.G., Baas F., Huitinga I., Ramaglia V. (2015). Complement C1q-C3-associated synaptic changes in multiple sclerosis hippocampus. Ann. Neurol..

[20] McHarg S., Clark S.J., Day A.J., Bishop P.N. (2015). Age-related macular degeneration and the role of the complement system. Mol. Immunol..

[21] Merle N.S., Grunenwald A., Rajaratnam H., Gnemmi V., Frimat M., Figueres M.-L., Knockaert S., Bouzekri S., Charue D., Noe R. (2018). Intravascular hemolysis activates complement via cell-free heme and heme-loaded microvesicles. JCI Insight.

[22] Sekar A., Bialas A.R., de Rivera H., Davis A., Hammond T.R., Kamitaki N., Tooley K., Presumey J., Baum M., Van Doren V. (2016). Schizophrenia risk from complex variation of complement component 4. Nature.

[23] Roumenina L.T., Daugan M.V., Noé R., Petitprez F., Vano Y.A., Sanchez-Salas R., Becht E., Meilleroux J., Clec’h B.L., Giraldo N.A. (2019). Tumor Cells Hijack Macrophage-Produced Complement C1q to Promote Tumor Growth. Cancer Immunol. Res..

[24] Ajona D., Ortiz-Espinosa S., Pio R. (2019). Complement anaphylatoxins C3a and C5a: Emerging roles in cancer progression and treatment. Semin. Cell Dev. Biol..

[25] Ajona D., Okrój M., Pajares M.J., Agorreta J., Lozano M.D., Zulueta J.J., Verri C., Roz L., Sozzi G., Pastorino U. (2018). Complement C4d-specific antibodies for the diagnosis of lung cancer. Oncotarget.

[26] Riihilä P., Nissinen L., Farshchian M., Kallajoki M., Kivisaari A., Meri S., Grénman R., Peltonen S., Peltonen J., Pihlajaniemi T. (2017). Complement Component C3 and Complement Factor B Promote Growth of Cutaneous Squamous Cell Carcinoma. Am. J. Pathol..

[27] Ajona D., Castaño Z., Garayoa M., Zudaire E., Pajares M.J., Martinez A., Cuttitta F., Montuenga L.M., Pio R. (2004). Expression of complement factor H by lung cancer cells: Effects on the activation of the alternative pathway of complement. Cancer Res..

[28] Riihilä P., Nissinen L., Farshchian M., Kivisaari A., Ala-Aho R., Kallajoki M., Grénman R., Meri S., Peltonen S., Peltonen J. (2015). Complement factor I promotes progression of cutaneous squamous cell carcinoma. J. Investig. Dermatol..

[29] Ravindranath N.M.H., Shuler C. (2006). Expression of complement restriction factors (CD46, CD55 & CD59) in head and neck squamous cell carcinomas. J. Oral Pathol. Med..

[30] Kapka-Skrzypczak L., Wolinska E., Szparecki G., Wilczynski G.M., Czajka M., Skrzypczak M. (2015). CD55, CD59, factor H and factor H-like 1 gene expression analysis in tumors of the ovary and corpus uteri origin. Immunol. Lett..

[31] Pio R., Corrales L., Lambris J.D. (2014). The role of complement in tumor growth. Adv. Exp. Med. Biol..

[32] Netti G.S., Lucarelli G., Spadaccino F., Castellano G., Gigante M., Divella C., Rocchetti M.T., Rascio F., Mancini V., Stallone G. (2020). PTX3 modulates the immunoflogosis in tumor microenvironment and is a prognostic factor for patients with clear cell renal cell carcinoma. Aging.

[33] Lucas S.D., Karlsson-Parra A., Nilsson B., Grimelius L., Akerström G., Rastad J., Juhlin C. (1996). Tumor-specific deposition of immunoglobulin G and complement in papillary thyroid carcinoma. Hum. Pathol..

[34] Kwak J.W., Laskowski J., Li H.Y., McSharry M.V., Sippel T.R., Bullock B.L., Johnson A.M., Poczobutt J.M., Neuwelt A.J., Malkoski S.P. (2018). Complement Activation via a C3a Receptor Pathway Alters CD4+ T Lymphocytes and Mediates Lung Cancer Progression. Cancer Res..

[35] Zirakzadeh A.A., Sherif A., Rosenblatt R., Ahlén Bergman E., Winerdal M., Yang D., Cederwall J., Jakobsson V., Hyllienmark M., Winqvist O. (2020). Tumour-associated B cells in urothelial urinary bladder cancer. Scand. J. Immunol..

[36] Wang Y., Zhang H., He Y.-W. (2019). The Complement Receptors C3aR and C5aR Are a New Class of Immune Checkpoint Receptor in Cancer Immunotherapy. Front. Immunol..

[37] Ajona D., Zandueta C., Corrales L., Moreno H., Pajares M.J., Ortiz-Espinosa S., Martínez-Terroba E., Perurena N., de Miguel F.J., Jantus-Lewintre E. (2018). Blockade of the Complement C5a/C5aR1 Axis Impairs Lung Cancer Bone Metastasis by CXCL16-mediated Effects. Am. J. Respir. Crit. Care Med..

[38] Markiewski M.M., DeAngelis R.A., Benencia F., Ricklin-Lichtsteiner S.K., Koutoulaki A., Gerard C., Coukos G., Lambris J.D. (2008). Modulation of the antitumor immune response by complement. Nat. Immunol..

[39] Nabizadeh J.A., Manthey H.D., Steyn F.J., Chen W., Widiapradja A., Md Akhir F.N., Boyle G.M., Taylor S.M., Woodruff T.M., Rolfe B.E. (2016). The Complement C3a Receptor Contributes to Melanoma Tumorigenesis by Inhibiting Neutrophil and CD4+ T Cell Responses. J. Immunol..

[40] Xu Y., Huang Y., Xu W., Zheng X., Yi X., Huang L., Wang Y., Wu K. (2020). Activated Hepatic Stellate Cells (HSCs) Exert Immunosuppressive Effects in Hepatocellular Carcinoma by Producing Complement C3. OncoTargets Ther..

[41] Davidson S., Efremova M., Riedel A., Mahata B., Pramanik J., Huuhtanen J., Kar G., Vento-Tormo R., Hagai T., Chen X. (2020). Single-Cell RNA Sequencing Reveals a Dynamic Stromal Niche That Supports Tumor Growth. Cell Rep..

[42] Jackson W.D., Gulino A., Fossati-Jimack L., Seoane R.C., Tian K., Best K., Köhl J., Belmonte B., Strid J., Botto M. (2020). C3 Drives Inflammatory Skin Carcinogenesis Independently of C5. J. Investig. Dermatol..

[43] Ding P., Li L., Li L., Lv X., Zhou D., Wang Q., Chen J., Yang C., Xu E., Dai W. (2020). C5aR1 is a master regulator in Colorectal Tumorigenesis via Immune modulation. Theranostics.

[44] Zha H., Wang X., Zhu Y., Chen D., Han X., Yang F., Gao J., Hu C., Shu C., Feng Y. (2019). Intracellular Activation of Complement C3 Leads to PD-L1 Antibody Treatment Resistance by Modulating Tumor-Associated Macrophages. Cancer Immunol. Res..

[45] Janelle V., Langlois M.-P., Tarrab E., Lapierre P., Poliquin L., Lamarre A. (2014). Transient complement inhibition promotes a tumor-specific immune response through the implication of natural killer cells. Cancer Immunol. Res..

[46] Vadrevu S.K., Chintala N.K., Sharma S.K., Sharma P., Cleveland C., Riediger L., Manne S., Fairlie D.P., Gorczyca W., Almanza O. (2014). Complement C5a Receptor Facilitates Cancer Metastasis by Altering T-Cell Responses in the Metastatic Niche. Cancer Res..

[47] Corrales L., Ajona D., Rafail S., Lasarte J.J., Riezu-Boj J.I., Lambris J.D., Rouzaut A., Pajares M.J., Montuenga L.M., Pio R. (2012). Anaphylatoxin C5a creates a favorable microenvironment for lung cancer progression. J. Immunol..

[48] Bonavita E., Gentile S., Rubino M., Maina V., Papait R., Kunderfranco P., Greco C., Feruglio F., Molgora M., Laface I. (2015). PTX3 is an extrinsic oncosuppressor regulating complement-dependent inflammation in cancer. Cell.

[49] Surace L., Lysenko V., Fontana A.O., Cecconi V., Janssen H., Bicvic A., Okoniewski M., Pruschy M., Dummer R., Neefjes J. (2015). Complement is a central mediator of radiotherapy-induced tumor-specific immunity and clinical response. Immunity.

[50] Gunn L., Ding C., Liu M., Ma Y., Qi C., Cai Y., Hu X., Aggarwal D., Zhang H.-G., Yan J. (2012). Opposing roles for complement component C5a in tumor progression and the tumor microenvironment. J. Immunol..

[51] Lu Y., Zhao Q., Liao J.-Y., Song E., Xia Q., Pan J., Li Y., Li J., Zhou B., Ye Y. (2020). Complement Signals Determine Opposite Effects of B Cells in Chemotherapy-Induced Immunity. Cell.

[52] Hong Q., Sze C.-I., Lin S.-R., Lee M.-H., He R.-Y., Schultz L., Chang J.-Y., Chen S.-J., Boackle R.J., Hsu L.-J. (2009). Complement C1q activates tumor suppressor WWOX to induce apoptosis in prostate cancer cells. PLoS ONE.

[53] Bandini S., Macagno M., Hysi A., Lanzardo S., Conti L., Bello A., Riccardo F., Ruiu R., Merighi I.F., Forni G. (2016). The non-inflammatory role of C1q during Her2/neu-driven mammary carcinogenesis. Oncoimmunology.

[54] Kaur A., Sultan S.H.A., Murugaiah V., Pathan A.A., Alhamlan F.S., Karteris E., Kishore U. (2016). Human C1q Induces Apoptosis in an Ovarian Cancer Cell Line via Tumor Necrosis Factor Pathway. Front. Immunol..

[55] Bulla R., Tripodo C., Rami D., Ling G.S., Agostinis C., Guarnotta C., Zorzet S., Durigutto P., Botto M., Tedesco F. (2016). C1q acts in the tumour microenvironment as a cancer-promoting factor independently of complement activation. Nat. Commun..

[56] Riihilä P.M., Nissinen L.M., Ala-Aho R., Kallajoki M., Grénman R., Meri S., Peltonen S., Peltonen J., Kähäri V.-M. (2014). Complement factor H: A biomarker for progression of cutaneous squamous cell carcinoma. J. Investig. Dermatol..

[57] Laskowski J., Renner B., Pickering M.C., Serkova N.J., Smith-Jones P.M., Clambey E.T., Nemenoff R.A., Thurman J.M. (2020). Complement factor H-deficient mice develop spontaneous hepatic tumors. J. Clin. Investig..

[58] Arbore G., Kemper C., Kolev M. (2017). Intracellular complement—The complosome—In immune cell regulation. Mol. Immunol..

[59] Ghebrehiwet B. (2020). Complement proteins in unexpected places: Why we should be excited, not concerned!. F1000Research.

[60] Lu Y., Hu X.-B. (2014). C5a stimulates the proliferation of breast cancer cells via Akt-dependent RGC-32 gene activation. Oncol. Rep..

[61] Cho M.S., Vasquez H.G., Rupaimoole R., Pradeep S., Wu S., Zand B., Han H.-D., Rodriguez-Aguayo C., Bottsford-Miller J., Huang J. (2014). Autocrine effects of tumor-derived complement. Cell Rep..

[62] Piao C., Cai L., Qiu S., Jia L., Song W., Du J. (2015). Complement 5a Enhances Hepatic Metastases of Colon Cancer via Monocyte Chemoattractant Protein-1-mediated Inflammatory Cell Infiltration. J. Biol. Chem..

[63] Nitta H., Wada Y., Kawano Y., Murakami Y., Irie A., Taniguchi K., Kikuchi K., Yamada G., Suzuki K., Honda J. (2013). Enhancement of human cancer cell motility and invasiveness by anaphylatoxin C5a via aberrantly expressed C5a receptor (CD88). Clin. Cancer Res..

[64] Rozanov D.V., Sikora S., Godzik A., Postnova T.I., Golubkov V., Savinov A., Tomlinson S., Strongin A.Y. (2004). Non-proteolytic, receptor/ligand interactions associate cellular membrane type-1 matrix metalloproteinase with the complement component C1q. J. Biol. Chem..

[65] Vlaicu S.I., Tegla C.A., Cudrici C.D., Danoff J., Madani H., Sugarman A., Niculescu F., Mircea P.A., Rus V., Rus H. (2013). Role of C5b-9 complement complex and response gene to complement-32 (RGC-32) in cancer. Immunol. Res..

[66] Carmeliet P. (2003). Angiogenesis in health and disease. Nat. Med..

[67] Nunez-Cruz S., Gimotty P.A., Guerra M.W., Connolly D.C., Wu Y.-Q., DeAngelis R.A., Lambris J.D., Coukos G., Scholler N. (2012). Genetic and pharmacologic inhibition of complement impairs endothelial cell function and ablates ovarian cancer neovascularization. Neoplasia.

[68] Kurihara R., Yamaoka K., Sawamukai N., Shimajiri S., Oshita K., Yukawa S., Tokunaga M., Iwata S., Saito K., Chiba K. (2010). C5a promotes migration, proliferation, and vessel formation in endothelial cells. Inflamm. Res..

[69] Bandini S., Curcio C., Macagno M., Quaglino E., Arigoni M., Lanzardo S., Hysi A., Barutello G., Consolino L., Longo D.L. (2013). Early onset and enhanced growth of autochthonous mammary carcinomas in C3-deficient Her2/neu transgenic mice. Oncoimmunology.

[70] Ying L., Zhang F., Pan X., Chen K., Zhang N., Jin J., Wu J., Feng J., Yu H., Jin H. (2016). Complement component 7 (C7), a potential tumor suppressor, is correlated with tumor progression and prognosis. Oncotarget.

[71] Rozenberg P., Ziporen L., Gancz D., Saar-Ray M., Fishelson Z. (2018). Cooperation between Hsp90 and mortalin/GRP75 in resistance to cell death induced by complement C5b-9. Cell Death Dis..

[72] Bouwens T.A.M., Trouw L.A., Veerhuis R., Dirven C.M.F., Lamfers M.L.M., Al-Khawaja H. (2015). Complement activation in Glioblastoma multiforme pathophysiology: Evidence from serum levels and presence of complement activation products in tumor tissue. J. Neuroimmunol..

[73] Zhao P., Wu J., Lu F., Peng X., Liu C., Zhou N., Ying M. (2019). The imbalance in the complement system and its possible physiological mechanisms in patients with lung cancer. BMC Cancer.

[74] Grzmil M., Voigt S., Thelen P., Hemmerlein B., Helmke K., Burfeind P. (2004). Up-regulated expression of the MAT-8 gene in prostate cancer and its siRNA-mediated inhibition of expression induces a decrease in proliferation of human prostate carcinoma cells. Int. J. Oncol..

[75] Oner F., Savaş I., Numanoğlu N. (2004). Immunoglobulins and complement components in patients with lung cancer. Tuberk Toraks.

[76] Lu Z.-L., Chen Y.-J., Jing X.-Y., Wang N.-N., Zhang T., Hu C.-J. (2018). Detection and Identification of Serum Peptides Biomarker in Papillary Thyroid Cancer. Med. Sci. Monit. Int. Med. J. Exp. Clin. Res..

[77] Ajona D., Razquin C., Pastor M.D., Pajares M.J., Garcia J., Cardenal F., Fleischhacker M., Lozano M.D., Zulueta J.J., Schmidt B. (2015). Elevated levels of the complement activation product C4d in bronchial fluids for the diagnosis of lung cancer. PLoS ONE.

[78] Kim P.Y., Tan O., Diakiw S.M., Carter D., Sekerye E.O., Wasinger V.C., Liu T., Kavallaris M., Norris M.D., Haber M. (2014). Identification of plasma complement C3 as a potential biomarker for neuroblastoma using a quantitative proteomic approach. J. Proteom..

[79] Chen J., Wu W., Chen L., Ma X., Zhao Y., Zhou H., Yang R., Hu L. (2014). Expression and clinical significance of AHSG and complement C3 in pancreatic ductal adenocarcinoma. Zhonghua Yi Xue Za Zhi.

[80] Chen J., Wu W., Zhen C., Zhou H., Yang R., Chen L., Hu L. (2013). Expression and clinical significance of complement C3, complement C4b1 and apolipoprotein E in pancreatic cancer. Oncol. Lett..

[81] Zhang X., Sun L. (2018). Anaphylatoxin C3a: A potential biomarker for esophageal cancer diagnosis. Mol. Clin. Oncol..

[82] Chung L., Moore K., Phillips L., Boyle F.M., Marsh D.J., Baxter R.C. (2014). Novel serum protein biomarker panel revealed by mass spectrometry and its prognostic value in breast cancer. Breast Cancer Res. BCR.

[83] Chen J., Li G.-Q., Zhang L., Tang M., Cao X., Xu G.-L., Wu Y.-Z. (2018). Complement C5a/C5aR pathway potentiates the pathogenesis of gastric cancer by down-regulating p21 expression. Cancer Lett..

[84] Helmig S., Lochnit G., Schneider J. (2019). Comparative proteomic analysis in serum of former uranium miners with and without radon induced squamous lung cancer. J. Occup. Med. Toxicol. Lond. Engl..

[85] Pio R., Garcia J., Corrales L., Ajona D., Fleischhacker M., Pajares M.J., Cardenal F., Seijo L., Zulueta J.J., Nadal E. (2010). Complement factor H is elevated in bronchoalveolar lavage fluid and sputum from patients with lung cancer. Cancer Epidemiol. Biomark. Prev. Publ. Am. Assoc. Cancer Res. Cosponsored Am. Soc. Prev. Oncol..

[86] Cheng Z.-Z., Corey M.J., Pärepalo M., Majno S., Hellwage J., Zipfel P.F., Kinders R.J., Raitanen M., Meri S., Jokiranta T.S. (2005). Complement factor H as a marker for detection of bladder cancer. Clin. Chem..

[87] Heicappell R., Müller M., Fimmers R., Miller K. (2000). Qualitative determination of urinary human complement factor H-related protein (hcfHrp) in patients with bladder cancer, healthy controls, and patients with benign urologic disease. Urol. Int..

[88] Narayanasamy A., Ahn J.-M., Sung H.-J., Kong D.-H., Ha K.-S., Lee S.-Y., Cho J.-Y. (2011). Fucosylated glycoproteomic approach to identify a complement component 9 associated with squamous cell lung cancer (SQLC). J. Proteom..

[89] Shang Y., Chai N., Gu Y., Ding L., Yang Y., Zhou J., Ren G., Hao X., Fan D., Wu K. (2014). Systematic immunohistochemical analysis of the expression of CD46, CD55, and CD59 in colon cancer. Arch. Pathol. Lab. Med..

[90] Swierzko A.S., Szala A., Sawicki S., Szemraj J., Sniadecki M., Sokolowska A., Kaluzynski A., Wydra D., Cedzynski M. (2014). Mannose-Binding Lectin (MBL) and MBL-associated serine protease-2 (MASP-2) in women with malignant and benign ovarian tumours. Cancer Immunol. Immunother. CII.

[91] Ytting H., Jensenius J.C., Christensen I.J., Thiel S., Nielsen H.J. (2004). Increased activity of the mannan-binding lectin complement activation pathway in patients with colorectal cancer. Scand. J. Gastroenterol..

[92] Li W., Liu Z., Liang B., Chen S., Zhang X., Tong X., Lou W., Le L., Tang X., Fu F. (2018). Identification of core genes in ovarian cancer by an integrative meta-analysis. J. Ovarian Res..

[93] Kim Y.-S., Hwan J.D., Bae S., Bae D.-H., Shick W.A. (2010). Identification of differentially expressed genes using an annealing control primer system in stage III serous ovarian carcinoma. BMC Cancer.

[94] Liu W., Liu B., Xin L., Zhang Y., Chen X., Zhu Z., Lin Y. (2007). Down-regulated expression of complement factor I: A potential suppressive protein for gastric cancer identified by serum proteome analysis. Clin. Chim. Acta Int. J. Clin. Chem..

[95] Lim L.C., Looi M.L., Zakaria S.Z.S., Sagap I., Rose I.M., Chin S.-F., Jamal R. (2016). Identification of Differentially Expressed Proteins in the Serum of Colorectal Cancer Patients Using 2D-DIGE Proteomics Analysis. Pathol. Oncol. Res. POR.

[96] Ricklin D., Mastellos D.C., Reis E.S., Lambris J.D. (2018). The renaissance of complement therapeutics. Nat. Rev. Nephrol..

[97] Ajona D., Ortiz-Espinosa S., Moreno H., Lozano T., Pajares M.J., Agorreta J., Bértolo C., Lasarte J.J., Vicent S., Hoehlig K. (2017). A Combined PD-1/C5a Blockade Synergistically Protects against Lung Cancer Growth and Metastasis. Cancer Discov..

[98] Diebolder C.A., Beurskens F.J., de Jong R.N., Koning R.I., Strumane K., Lindorfer M.A., Voorhorst M., Ugurlar D., Rosati S., Heck A.J.R. (2014). Complement is activated by IgG hexamers assembled at the cell surface. Science.

[99] Brahmer J., Reckamp K.L., Baas P., Crinò L., Eberhardt W.E.E., Poddubskaya E., Antonia S., Pluzanski A., Vokes E.E., Holgado E. (2015). Nivolumab versus Docetaxel in Advanced Squamous-Cell Non-Small-Cell Lung Cancer. N. Engl. J. Med..

[100] Zha H., Han X., Zhu Y., Yang F., Li Y., Li Q., Guo B., Zhu B. (2017). Blocking C5aR signaling promotes the anti-tumor efficacy of PD-1/PD-L1 blockade. Oncoimmunology.

[101] Massard C., Cassier P., Bendell J.C., Marie D.B., Blery M., Morehouse C., Ascierto M., Zerbib R., Mitry E., Tolcher A.W. (2019). Preliminary results of STELLAR-001, a dose escalation phase I study of the anti-C5aR, IPH5401, in combination with durvalumab in advanced solid tumours. Ann. Oncol..

[102] Afshar-Kharghan V. (2017). The role of the complement system in cancer. J. Clin. Investig..

[103] Zhang R., Liu Q., Li T., Liao Q., Zhao Y. (2019). Role of the complement system in the tumor microenvironment. Cancer Cell Int..

[104] Arbore G., West E.E., Rahman J., Le Friec G., Niyonzima N., Pirooznia M., Tunc I., Pavlidis P., Powell N., Li Y. (2018). Complement receptor CD46 co-stimulates optimal human CD8+ T cell effector function via fatty acid metabolism. Nat. Commun..

[105] Taylor R.P., Lindorfer M.A. (2016). Cytotoxic mechanisms of immunotherapy: Harnessing complement in the action of anti-tumor monoclonal antibodies. Semin. Immunol..

[106] Coiffier B., Haioun C., Ketterer N., Engert A., Tilly H., Ma D., Johnson P., Lister A., Feuring-Buske M., Radford J.A. (1998). Rituximab (anti-CD20 monoclonal antibody) for the treatment of patients with relapsing or refractory aggressive lymphoma: A multicenter phase II study. Blood.

[107] Zent C.S., Secreto C.R., LaPlant B.R., Bone N.D., Call T.G., Shanafelt T.D., Jelinek D.F., Tschumper R.C., Kay N.E. (2008). Direct and complement dependent cytotoxicity in CLL cells from patients with high-risk early-intermediate stage chronic lymphocytic leukemia (CLL) treated with alemtuzumab and rituximab. Leuk. Res..

[108] Van de Donk N.W.C.J., Janmaat M.L., Mutis T., Lammerts van Bueren J.J., Ahmadi T., Sasser A.K., Lokhorst H.M., Parren P.W.H.I. (2016). Monoclonal antibodies targeting CD38 in hematological malignancies and beyond. Immunol. Rev..

[109] Lee C.-H., Romain G., Yan W., Watanabe M., Charab W., Todorova B., Lee J., Triplett K., Donkor M., Lungu O.I. (2017). IgG Fc domains that bind C1q but not effector Fcγ receptors delineate the importance of complement-mediated effector functions. Nat. Immunol..

[110] Middleton O., Cosimo E., Dobbin E., McCaig A.M., Clarke C., Brant A.M., Leach M.T., Michie A.M., Wheadon H. (2015). Complement deficiencies limit CD20 monoclonal antibody treatment efficacy in CLL. Leukemia.

[111] Dzietczenia J., Wróbel T., Mazur G., Poreba R., Jaźwiec B., Kuliczkowski K. (2010). Expression of complement regulatory proteins: CD46, CD55, and CD59 and response to rituximab in patients with CD20+ non-Hodgkin’s lymphoma. Med. Oncol. Northwood Lond. Engl..

[112] Pawluczkowycz A.W., Beurskens F.J., Beum P.V., Lindorfer M.A., van de Winkel J.G.J., Parren P.W.H.I., Taylor R.P. (2009). Binding of submaximal C1q promotes complement-dependent cytotoxicity (CDC) of B cells opsonized with anti-CD20 mAbs ofatumumab (OFA) or rituximab (RTX): Considerably higher levels of CDC are induced by OFA than by RTX. J. Immunol..

[113] Cook E.M., Lindorfer M.A., van der Horst H., Oostindie S., Beurskens F.J., Schuurman J., Zent C.S., Burack R., Parren P.W.H.I., Taylor R.P. (2016). Antibodies That Efficiently Form Hexamers upon Antigen Binding Can Induce Complement-Dependent Cytotoxicity under Complement-Limiting Conditions. J. Immunol..

[114] Oostindie S.C., van der Horst H.J., Lindorfer M.A., Cook E.M., Tupitza J.C., Zent C.S., Burack R., VanDerMeid K.R., Strumane K., Chamuleau M.E.D. (2019). CD20 and CD37 antibodies synergize to activate complement by Fc-mediated clustering. Haematologica.

[115] Pedersen D.V., Rösner T., Hansen A.G., Andersen K.R., Thiel S., Andersen G.R., Valerius T., Laursen N.S. (2020). Recruitment of properdin by bi-specific nanobodies activates the alternative pathway of complement. Mol. Immunol..

[116] Gelderman K.A., Lam S., Sier C.F., Gorter A. (2006). Cross-linking tumor cells with effector cells via CD55 with a bispecific mAb induces beta-glucan-dependent CR3-dependent cellular cytotoxicity. Eur. J. Immunol..

[117] Cruz J.W., Damko E., Modi B., Tu N., Meagher K., Voronina V., Gartner H., Ehrlich G., Rafique A., Babb R. (2019). A novel bispecific antibody platform to direct complement activity for efficient lysis of target cells. Sci. Rep..

[118] Ajona D., Hsu Y.-F., Corrales L., Montuenga L.M., Pio R. (2007). Down-regulation of human complement factor H sensitizes non-small cell lung cancer cells to complement attack and reduces in vivo tumor growth. J. Immunol..

[119] Petitprez F., de Reyniès A., Keung E.Z., Chen T.W.-W., Sun C.-M., Calderaro J., Jeng Y.-M., Hsiao L.-P., Lacroix L., Bougoüin A. (2020). B cells are associated with survival and immunotherapy response in sarcoma. Nature.

[120] Ajona D., Pajares M.J., Corrales L., Perez-Gracia J.L., Agorreta J., Lozano M.D., Torre W., Massion P.P., de-Torres J.P., Jantus-Lewintre E. (2013). Investigation of complement activation product c4d as a diagnostic and prognostic biomarker for lung cancer. J. Natl. Cancer Inst..

